# Molecular Modulation of Fetal Liver Hematopoietic Stem Cell Mobilization into Fetal Bone Marrow in Mice

**DOI:** 10.1155/2020/8885154

**Published:** 2020-12-15

**Authors:** Huihong Zeng, Jiaoqi Cheng, Ying Fan, Yingying Luan, Juan Yang, Feixuan Wang, Shuo Yang, Lijian Shao

**Affiliations:** ^1^Medical College of Nanchang University, Nanchang, China 330006; ^2^Jiangxi Provincial Key Laboratory of Preventive Medicine, Nanchang University, Nanchang, China 330006

## Abstract

Development of hematopoietic stem cells is a complex process, which has been extensively investigated. Hematopoietic stem cells (HSCs) in mouse fetal liver are highly expanded to prepare for mobilization of HSCs into the fetal bone marrow. It is not completely known how the fetal liver niche regulates HSC expansion without loss of self-renewal ability. We reviewed current progress about the effects of fetal liver niche, chemokine, cytokine, and signaling pathways on HSC self-renewal, proliferation, and expansion. We discussed the molecular regulations of fetal HSC expansion in mouse and zebrafish. It is also unknown how HSCs from the fetal liver mobilize, circulate, and reside into the fetal bone marrow niche. We reviewed how extrinsic and intrinsic factors regulate mobilization of fetal liver HSCs into the fetal bone marrow, which provides tools to improve HSC engraftment efficiency during HSC transplantation. Understanding the regulation of fetal liver HSC mobilization into the fetal bone marrow will help us to design proper clinical therapeutic protocol for disease treatment like leukemia during pregnancy. We prospect that fetal cells, including hepatocytes and endothelial and hematopoietic cells, might regulate fetal liver HSC expansion. Components from vascular endothelial cells and bones might also modulate the lodging of fetal liver HSCs into the bone marrow. The current review holds great potential to deeply understand the molecular regulations of HSCs in the fetal liver and bone marrow in mammals, which will be helpful to efficiently expand HSCs *in vitro*.

## 1. Introduction

Hematopoietic stem cells (HSCs) differentiate into mature cells through multiple types of progenitors, which maintain lifelong blood cell generation [1]. Self-renewal and differentiation capacities of HSCs were extensively investigated. Several types of leukemia were effectively treated through hematopoietic stem cell transplantation [2]. However, limited numbers of available HSCs impede their clinical practice. *In vitro* HSC expansion, embryonic stem cell- and induced pluripotent stem cell-derived hematopoietic progenitors were extensively investigated. Most expanded hematopoietic stem and progenitor cells easily lose their self-renewal and differentiation abilities *in vivo* [3, 4]. The dilemma indicates that there are functional and molecular gaps between expanded HSCs and well-developed HSCs.

During hematopoietic development, fetal hematopoietic stem cells migrate into different microenvironments to acquire special molecular properties, which assist HSC expansion, migration, homing, and finally engraftment into the bone marrow (in mammals) or the kidney (in zebrafish) [5]. However, the exact origin of the developmental of long-term HSCs is holding long-term controversial. Mikkola et al. extensively reviewed the journey of developmental HSCs in 2006 [6, 7]. The origin of primitive hematopoiesis in mammals might be derived from intraembryonic AGM (aorta-gonad mesonephros) region and extraembryonic sites (yolk sac and placenta) during the early developmental period [7, 8]. Hematopoietic stem cells are derived from hemogenic endothelial (HE) cells, which reside in the dorsal aorta (in mammals) or the caudal artery (in zebrafish). Subsequently, HE cells experience an endothelial cell to hematopoietic cell transition (EHT) that generates HSCs in the intra-arterial clusters (IAC). Due to the rapid development of single-cell analysis technology, numbers of studies have examined the molecular mechanisms that mediate the transition from HE to EHT to IAC through single-cell RNA sequencing (scRNA-Seq) and single-cell assay for transposase-accessible chromatin sequencing (scATAC-Seq) [9–12]. These technologies help to establish a continuous developmental trajectory at the molecular level during hematopoietic development. Most recently, how microenvironment regulates HSC during development was extensively studied using bulk RNA-Seq, scRNA-Seq, CHIP-Seq techniques, and so on. For example, Laurent et al. used genome-wide RNA tomography sequencing to explore the complexity of the aortic microenvironment and various factors interacting to control hematopoietic stem cell generation in multispecies [10]. They found that knocking down of either ADM or its receptor RAMP2 significantly decreased HSC production in zebrafish. The roles of secreted growth factors from the microenvironment on HSC development were also investigated, showing that deletion of SVEP1 negatively affected the engraftment of HSCs upon transplantation [10]. Gao et al. investigated the transcriptional regulatory network during the ontogeny of HSCs in mouse embryos. They used RNA-Seq and ChIP-Seq techniques to define the transcriptomes and epigenomes of HSC ontogeny, demonstrating that 78% of enhancers are active at the early developmental stage [11]. The functional importance of transcription factors SP3 and Myc-associated zinc finger (MAZ) was validated during the formation of hemogenic endothelium in zebrafish. These data indicate that mutations of SP3 and MAZ significantly reduced numbers of HE and HSCs [11]. Frame et al. demonstrated that NLRP3 inflammasome-mediated interleukin-1-beta (IL-1*β*) signaling positively regulated HSC production in response to metabolic activity in zebrafish [13]. These data imply that there is a complex interaction between the microenvironment and HSCs during the early developmental stage.

Regardless of the origin of hematopoietic stem cells, primitive hematopoietic stem and progenitors eventually migrate into fetal livers from either the AGM or yolk sac or placenta starting at embryonic day 11.5 (E11.5) in mice [7, 14]. In zebrafish, HSCs migrate into the caudal hematopoietic tissue (CHT), the counterpart of the mammalian fetal liver, for expansion and differentiation. The fetal liver HSCs were significantly expanded up to 40-fold from E11.5 to E14.5 in mouse, which was modulated by different signaling pathways, such as Notch and Wnt signaling pathways [6, 7, 15, 16]. Starting from E15.5, expanded mouse fetal liver HSCs gradually migrate into the blood, spleen, and bone marrow [17]. They finally reside into the fetal bone marrow in mice, supplying hematopoietic cells during life. However, how the microenvironment in the fetal liver or CHT regulates HSC expansion remains elusive. What molecular mechanisms regulate the migration journey of fetal HSCs into the bone marrow is ill-defined.

Even though a number of known factors or signaling pathways are crucial for functional fetal HSCs, investigations of the molecular mechanisms of fetal liver HSC mobilization into the fetal bone marrow are rarely seen. Additionally, the migration process of fetal liver HSCs into the bone marrow is a perfect *in vivo* model to explore the molecular regulation of HSC homing and engraftment. In this *de novo* model, both HSCs and niche are in physiological and undisturbed conditions. However, either transplanted HSCs or recipient niche is negatively stressed by chemicals or irradiation in the current popular clinical practice for the bone marrow transplantation.

Understanding the molecular modulation of fetal liver HSC migration into the bone marrow is of significance to properly manipulate and evaluate *in vitro* HSC expansion. In the current review, we will discuss some known mediators that regulate fetal liver HSC expansion, migration, and colonization into the bone marrow. Underpinning molecular mechanisms were reviewed during the migration of fetal liver HSCs into the fetal bone marrow. In addition, migration and regulation of primitive hematopoietic stem cells from either the AGM or yolk sac or placenta into the fetal liver will not be further discussed here because they have been extensively reviewed by others [5, 18].

## 2. Regulation of Fetal Liver Hematopoietic Stem Cells

In the fetal liver, HSCs from the AGM or yolk sac or placenta undergo extreme expansion between E12.5 and E15.5 in mouse [16]. Fetal liver HSCs are active and proliferate with symmetric and asymmetric division [19]. In contrast, adult HSCs are usually predominant and quiescent in the bone marrow [20]. An adult HSC experiences asymmetric division generating one progenitor and one HSC, leading to maintenance of HSC numbers throughout life. The distinguished characteristic of fetal and adult HSCs might contribute to (1) their different specialized microenvironments and HSC niches and (2) different molecular properties. Here, we discuss fetal liver HSCs and niches mainly using mouse strain as our example.

### 2.1. Fetal Liver HSC Niches (Figure 1)

In the mouse fetal liver, the microenvironment of hematopoietic stem cells mainly consists of hepatoblasts and endothelial and stromal cells. This is different from adult HSC niches, which are mainly composed of two types of niches, the osteoblastic niche and vascular niche [21]. Recently, mesenchymal stromal cells in the adult bone marrow, such as Leptin receptor^+^, Nestin^+^, and NG2^+^ cells, produce multiple factors to affect HSC features positively or negatively. Most likely, components from fetal niches stimulate the rapid expansion of functional HSCs in the mouse fetal liver from E12.5 to E15.5. Fetal liver HSCs at E14.5 undergo a dramatic expansion *in vivo* and have stronger engraftment potential than adult HSCs from the bone marrow. However, the efficient expansion of fetal liver HSCs has failed *in vitro*, suggesting that fetal liver environment-derived factors are crucial during HSC expansion. This concept was supported by the study from Choong et al.'s group [22]. Fetal liver CD3^+^ cells, but not B220^+^, CD11b^+^, and Gr-1^+^ cells, efficiently expand functional fetal and adult HSCs. Further data showed that fetal liver CD3^+^ cells produced insulin-like growth factor 2 (IGF-2), which is able to enhance the expansion of both fetal liver and bone marrow HSCs [23]. Subsequent studies demonstrated that fetal liver CD3^+^ cell-derived angiopoietin-like 2 (Angptl2) and Angptl3 remarkably stimulated *ex vivo* expansion of fetal HSCs [24, 25]. These CD3^+^ cells were further identified as SCF^+^DLK^+^ population with fetal hepatic stem cell property. Fetal SCF^+^DLK^+^ population produces not only IGF2, Angptl2, and Angptl3 but also thrombopoietin (TPO), SDF-1*α*, and *α*-fetoprotein. These data indicate that fetal SCF^+^DLK^+^ stromal cells can produce multiple factors to stimulate HSC expansion in the mouse fetal liver [26]. Excepting SCF^+^DLK^+^ hepatic stem cell in the fetal liver, fetal endothelial cells can secrete SDF-1*α* chemokine to promote HSC maintenance and mobilization. Interestingly, bile acids, the active components of bile from the maternal and fetal liver, could expand fetal liver HSCs through inhibiting endoplasmic reticulum (ER) stress and protein aggregation [27].

EPCR^+^ HSCs in the fetal liver localize around the Lyve-1^+^ sinusoidal network through immunostaining and imaging, suggesting that the perisinusoidal network might be a niche for fetal liver HSCs in mouse [28]. Even though much less is known about fetal liver niche, fetal liver-derived stromal cell lines were previously reported to support HSC expansion *in vitro*. Existence of HSC niche in the fetal liver is supported by a recent study from Dr. Frenette's group [29, 30]. Using a genetic approach, they elegantly demonstrated that Nestin^+^NG2^+^ pericytes were associated with portal vessels, which form a niche in the mouse fetal liver. Nestin^+^NG2^+^ pericytes drive fetal HSC expansion with increasing cell cycle genes like Ki-67. The functional role of Nestin^+^NG2^+^ pericytes on fetal HSC expansion is independent of the production of SCF, Angptl2, and IGF2 [29, 30]. The numbers of Nestin^+^NG2^+^ cells in the mouse fetal liver are logarithmically increased from E12.5 to E14.5 [29, 30], which is consistent with fetal HSC expansion during the period. The expansion of Nestin^+^NG2^+^ pericytes around portal vessels is thus synchronized with HSC expansion. However, loss of Nestin^+^NG2^+^ cells at E12.5 does not affect HSC homing property, pointing out the specificity of Nestin^+^NG2^+^ cell niche for the fetal liver HSCs. In the circumstance of *in vivo* Nestin^+^NG2^+^ cell depletion, numbers of fetal liver HSCs were significantly reduced when compared to the controls [29, 30]. This is because deletion of Nestin^+^NG2^+^ cells significantly decreased proliferation of fetal liver HSCs, displaying that Nestin^+^NG2^+^ cells are required for fetal liver HSC proliferation and expansion in mouse.

Going back to look at the journey of developing HSCs, we realize that arterioles are tightly associated with hematopoietic stem cells at various developmental sites in mouse. In the AGM stage, primitive hematopoietic stem cells are attached to the largest artery aorta [31]. In the fetal liver, Nestin^+^NG2^+^ cells around arterial portal vessels are associated with fetal HSCs, which promote HSC proliferation without loss of self-renewal ability [30]. In the adult bone marrow, arterioles associated Nestin^+^NG2^+^ cells close to adult HSCs in the bone marrow, which can maintain HSC quiescence [32]. Therefore, the arterial vasculature may provide an adaptive microenvironment that supports hematopoiesis at various stages of mammalian life.

During the formation of the caudal vein plexus (CVP) in zebrafish, investigators found that HSCs in the CHT remodeled the neighboring endothelial cells (ECs) and stromal cells to form EC “pockets,” which facilitate HSC lodgment and expansion [33, 34]. Xue et al. used RNA-Seq technology to find that Kruppel-like factor 6a (Klf6a) is an essential component in the CHT endothelial niche. They demonstrated that Klf6a directly regulated expression of the chemokine ligand 25b (Ccl25b) in endothelial cells, which modulates HSC lodgment and proliferation [33]. Ccl25b from endothelial cells can bind to Ccr7 on the HSCs. The formation of Ccl25b/Ccr7 complex stimulates HSC proliferation and increases *in vitro* colony-forming ability of HSCs in both zebrafish and mouse. Therefore, Klf6a-Ccl25b/Ccr7 axis controls the HSC-CHT endothelial niche interaction and promotes HSC proliferation and expansion [33]. To further address how the CHT niche facilitates HSC expansion, the extrinsic factors involved in the dynamic developmental process of HSC expansion and differentiation were deeply analyzed [35]. The results show that many signaling pathways regulate niche-HSC interaction in zebrafish, such as Notch, BMP, and integrin signaling pathways [35]. Numbers of HSCs were significantly increased when expression of integrin *β*2 and its ligand Ctgfa was downregulated in zebrafish. These data indicate that the Ctgfa-integrin *β*2 signaling pathway has a negative role on the HSC expansion in the CHT in zebrafish [35]. However, it is unknown whether inhibiting the Ctgfa-integrin *β*2 signaling pathway stimulates HSC proliferation and expansion in mouse. Taken together, these data suggest that both intrinsic factors and niche-HSC interaction contribute to rapid expansion of HSCs in zebrafish.

It is unknown how hepatocytic cytokines, such as Aglptl3 and IGF2, are regulated by transcriptional factors in the fetal liver. Recently, Zhao et al.'s group demonstrated that activating transcription factor 4 (ATF4) was a potent positive regulator for the functional expansion and repopulation of HSCs in the mouse fetal liver but not in the AGM [36]. The results indicate that HSC functional defect under ATF4 deletion is noncell autonomous effects. It is because deletion of ATF4 leads to the disruption of the mouse fetal liver niche, along with the decrease of several cytokines such as Angptl3, VEGFA, and IGF2. The defect of those cytokines in the fetal liver causes hematopoietic stem cell defect. Angptl3 treatment can partially rescue the fetal HSC phenotype in ATF4-deficient embryos. It is unknown whether ATF4 deletion affects numbers of Nestin^+^NG2^+^ cells in the fetal liver, which might contribute to the functional defect of ATF4 knockout HSCs. Therefore, there are multiple niche components affecting hematopoietic stem cell development in the mouse fetal liver.

### 2.2. Fetal Liver HSCs

During hematopoietic ontogeny, fetal hematopoietic stem and progenitors migrate and circulate into different embryonic tissues in mammals, such as the AGM, yolk sac, placenta, liver, and bone marrow. Transplanted HSCs through mouse tail vein home into extravascular hematopoietic sites like the bone marrow in a few minutes to hours after transplantation, which might be mediated by adhesion molecules, such as CD18 and Pecam-1 [37]. In the transplantation setting, those transplanted HSCs rapidly home to different hematopoietic tissues, which recapitulate the physiological process in the fetal life. However, there are some fundamental differences between transplantation setting and real fetal life. In the case of adult HSCs, those HSCs in the G0/G1 phase of the cell cycle have optimal homing capacities [38]. Therefore, the homing ability of adult HSCs will be dramatically reduced after HSCs are exposed to various cytokines. In contrast, in the case of fetal HSCs, fetal HSCs in S+G2/M phases of the cell cycle can efficiently home and engraft into the irradiated recipient mice. Therefore, fetal HSCs are intrinsically different from adult HSCs in the migration and homing behavior. Xue et al.'s group took advantage of the scRNA-Seq technique to analyze gene expression profile in the different developmental stages of HSCs in zebrafish [35]. Their data showed that HSC in CHT in zebrafish highly expressed those genes enriched for the terms “rNRA processing,” “mRNA splicing,” and “ribosome biogenesis.” These data suggest that HSCs experience rapid expansion in CHT in zebrafish. Gao et al.'s group used transcriptome dynamic analysis during HSC ontogeny in mouse. The data showed that genes that peak in pre-HSCs were enriched for regulation of cell cycle and genes that peak in mouse fetal liver HSCs were enriched for functional HSC terms [11]. Collectively, fetal liver HSCs in mouse have all gene elements for their proliferation, expansion, and migration.

Adhesion molecules play crucial roles during the journey of mouse HSCs in different sites. Deletion of ESAM (endothelial cell-selective adhesion molecule) results in 50% fetal lethality during mid to later gestation in mice. The embryonic death occurs between E15.5 and E17.5, which is the time window of the migration of fetal liver HSCs into the fetal bone marrow in mouse [39, 40]. Further studies showed that the definitive HSCs in ESAM^−/−^ fetal livers were fewer than those in wild-type fetal livers. ESAM-deficient fetal HSCs failed to regulate erythropoiesis and synthesize adult-type hemoglobin due to downregulation of Hba, Hbb-1, and Alas2 expression. Therefore, maintaining ESAM expression is important for the maintenance and proliferation of fetal liver HSCs. CD44 is expressed in most mammalian cells including HSCs. CD44 expression plays a critical role in the adult hematopoiesis [41]. In adult mice, loss of CD44 increases the retention of hematopoietic progenitors in the BM and spleen. During hematopoietic development, the absence of CD44 results in increased frequency and number of HSCs in the fetal liver, which is accompanied by a delayed migration of HSCs from the fetal liver. These might be related to the changes of SDF-1*α* concentration with CD44 loss. SDF-1*α* concentration in wild-type fetal liver significantly decreased from E14.5 to E17.5, while there were no changes during the period in CD44^−/−^ fetal livers. Persistence of high concentration of SDF-1*α* in CD44^−/−^ fetal livers will delay the migration of fetal liver HSCs into the bone marrow, blood, and spleen.

N-cadherin (N-cad) expression was detected in E12.5 fetal liver HSCs, which are colocalized with sinusoidal endothelial cells [42]. N-cad^+^ HSCs in E12.5 fetal livers have higher engraftment ability in a transplantation assay when compared to N-cad^−^ HSCs. However, the level of N-cadherin expression starts to decrease in the E15.5 fetal liver, which promotes the detachment of N-cad^+^ HSCs from perisinusoidal niche [43]. These data suggest that the level of N-cadherin expression in fetal liver HSCs determines when HSCs will begin to mobilize out of the fetal liver despite the existing mixed results which are available for the effects of N-cadherin on adult HSCs [44, 45]. Taken together, the expression of adhesion molecules on HSCs tailors the critical migration window from the fetal liver into the bone marrow in mouse.

Xue et al. deciphered the molecular property of HSCs in zebrafish through bulk RNA-Seq and scRNA-Seq and identified 40 HSC-related genes, such as Smchd1 [35]. Smchd1 is an epigenetic regulator and encodes structural maintenance of chromosome flexible hinge-domain containing 1. Downregulation of Smchd1 significantly decreased numbers of HSCs in the CHT region in zebrafish. Further data indicated that decreased expression of Smchd1 reduced HSC proliferation and expansion [35]. Moreover, they found that HSCs and progenitors are in the G2-M phase and lymphoid and myeloid progenitors are in the G1 phase according to the cell cycle analysis from RNA-Seq data [35]. These data indicate that cell cycle features are associated with HSC differentiation.

**Figure 2:**
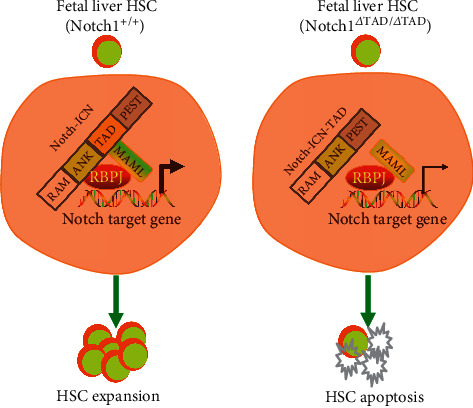
Notch1 transcriptional activation domain is essential for HSC expansion in the mouse fetal liver. Upon Notch1 activation, Notch1 intracellular domain (Notch1-ICN) gets into the nucleus forming complex with RBPJ and MAML, which increases expression of Notch1 downstream target genes and fetal HSC expansion. Loss of Notch1 transcriptional activation domain (Notch1*Δ*TAD/*Δ*TAD) leads to the failure of formation of Notch1-ICN/RBPJ/MAML transcription complex, which decreases the expression of Notch1 downstream target genes, resulting in HSC apoptosis.

In early embryonic hematopoietic stem cell development, Notch1-deficient embryos die in the early gestation [46]. It is thus difficult to investigate the Notch1 function in mid to later gestation using Notch1-deficient embryos. Recently, Gerhardt et al. found that Notch1 transcriptional activation domain- (Nothc1-TAD-) deficient mouse embryos can survive until late gestation (E18.5). Notch1 with transcriptional activation domain deletion can decrease Notch1 signaling with increasing fetal liver HSC apoptosis in mice. This leads to the decreased numbers of fetal HSCs, along with HSC functional impairment by competitive transplantation assay. Recently, we investigated the effects of losing one copy of Notch1-TAD (Notch1*Δ*/TAD) on the endothelial niche in the adult bone marrow. Our data displayed that the recovery of the endothelial niche in Notch1*Δ*/TAD mice was failed after chemotherapy and radiotherapy. We further proved that angiopoietin 1 activated Tie2 signaling, which results in Notch1 signaling activation and its target gene expression [47]. Mechanistically, loss of Notch1-TAD leads to the failure of properly assembling the Notch1/RBPJ/MAML trimolecular transcriptional complex, which results in decreased expression of Notch1 downstream targets, such as Hes1 and Dtx1 [46, 48]. Together, these studies reveal an essential role for the Notch1-TAD in fetal development and identify an important cell-autonomous function for Notch1 signaling in fetal HSCs (Figure 2). However, it is unknown whether cytokines from the fetal liver niche can activate Notch1 signaling in HSCs through Tie2 signaling activation. This might benefit HSC expansion in the fetal liver. The importance of Notch1 signaling on functional fetal liver HSCs was also observed in Cited2 (cAMP-responsive element binding protein [CBP]/p300-interacting transactivators with glutamic acid [E] and aspartic acid [D]–rich tail 2) knockout mouse embryos. Loss of Cited2 results in decreased numbers of fetal liver HSCs with the impairment of *in vivo* HSC engraftment, which is related to the reduction of Notch1 signaling [49].

It was documented that *β*-catenin/canonical Wnt signaling was important for HSC emergence, but not maintenance [50, 51]. The importance of *β*-catenin/canonical Wnt signaling on HSC function was determined using genetic deletion of *β*-catenin under the control with either VE-cadherin-Cre or Vav1-Cre [52, 53]. How Wnt signaling regulates the behavior of fetal liver HSCs was recently investigated. Kwarteng et al. used inducible Vav1-Cre to block *β*-catenin/canonical Wnt signaling in hematopoietic cells [19]. Their data suggest that fetal liver HSCs were more reliant on canonical *β*-catenin-dependent Wnt signaling than that in adult HSCs. Furthermore, *β*-catenin-dependent Wnt signaling protects fetal liver HSCs from oxidative stress under competitive transplantation condition [19]. Deaminase ADAR1 is essential for the maintenance of fetal liver HSCs via inhibiting cellular apoptosis and type I and II interferon signaling [54]. Using single-cell RNA-Seq, Zhou et al. showed that mTORC2 signaling was activated in embryonic hematopoietic stem cells [55]. The data from blocking mTORC2 signaling in endothelial cells imply that mTORC2 signaling is required for the generation of fetal HSCs but not hematopoietic progenitor cells. However, disruption of mTORC2 signaling in hematopoietic cells does not affect multilineage differentiation of fetal HSCs despite the fact that loss of mTORC2 signaling mildly reduced fetal HSC reconstitution ability [55, 56]. Therefore, multiple signaling pathways are involved in the fetal liver HSCs to accommodate the requirement of the developing hematopoietic cells.

## 3. Regulation of Fetal Bone Marrow Hematopoietic Stem Cells

Starting from E15.5 in mice, HSCs gradually migrate out fetal liver toward the spleen and bone marrow. Mobilized HSCs in the spleen have a limited proliferation capacity for unknown reasons. HSCs in the fetal bone marrow will successfully adapt to their new environment, sustaining lifelong hematopoietic cell generation. However, little is known about how the fetal bone marrow niche is formed and how the local microenvironment maintains stem cell properties. Although adult bone marrow niches were extensively investigated for decades, including the osteoblastic niche and endothelial niche [57], little is known about the formation of stem cell niches in the fetal bone marrow. It is critical for our understanding of normal hematopoiesis to identify components that can generate, maintain, and affect the HSC niche in the fetal bone marrow. It will be of significance for understanding of stem cell homing, migration, and lodgment to study stem cell differentiation and hematopoietic pathology.

**Figure 3:**
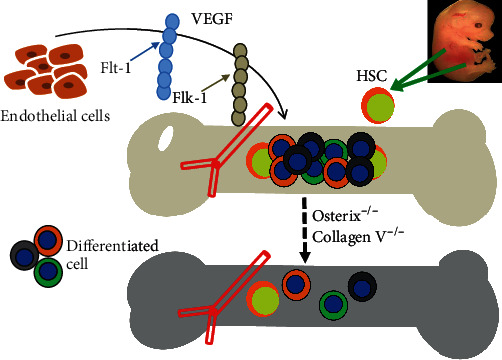
Endochondral ossification and vessel formation are required for lodgment of fetal liver HSC into the fetal bone marrow. Consistent levels of VEGF benefit vascularization in the fetal bone via binding with Flt-1 and Flk-1 receptors in endothelial cells. Vascularization in the fetal bone provides nutrition and aid niche formation. Loss of Osterix and collagen X negatively affects fetal liver HSC mobilization into the bone marrow through disrupting endochondral ossification.

Fetal bones consist of cartilages, vessels, and undetermined stromal cells. There are two types of bone formation, endochondral ossification and intramembranous ossification (Figure 3). It was proved that endochondral ossification is very important for the immediate setting down of mobilized fetal HSCs [58]. Therefore, hematopoietic development and bone formation are closely linked together at mid to later gestation. Many factors are involved in osteogenesis, such as CBFa1 and collagen X. For example, CBFa1-deficient mice lack both intramembranous and endochondral bone formation, which results in a complete lack of the bone marrow cavity in the entire skeleton. Normal numbers of hematopoietic precursors were observed in the CBFa1-deficient fetal liver before E17.5. After E18.5, large hematopoietic foci were observed in the liver and spleen but not in bones [59]. These data indicate that congenital lack of bone marrow causes excessive levels of extramedullary hematopoiesis in both the liver and spleen at the late embryonic stage. Growth plate compressions and hematopoietic aplasia were seen in collagen X knockout mice [60], implying that the disruption of collagen X function results in the skeleton-hematopoietic defects (Figure 3). These results suggest that endochondral osteogenesis and hypertrophic cartilage might contribute to the marrow environment for blood cell homeostasis.

Chan et al.'s group reported that CD105^+^Thy1^−^ cells from E15.5 fetal bones produce ectopic bones through a cartilage intermediate and generate a marrow cavity in the mouse kidney capsule [58, 61]. The ectopic bones recruit host-derived blood vessels with host-derived long-term reconstituting HSCs. Osterix knockdown severely inhibited osteogenesis and abolished niche formation [58]. Thus, endochondral ossification is involved in bone marrow niche formation. Endochondral ossification is associated with vascular invasion. Perichondral cells and chondrocytes express high levels of VEGF in the developing limb [62]. It was found that endochondral ossification was disrupted when fetal bones at E13.5 were transplanted into hosts with VEGF inhibition. The data indicates that VEGF activity is required for bone marrow niche formation. VEGF is a major regulator of blood vessel formation and hematopoiesis. VEGFR-1 and 2 can rescue survival and repopulation of VEGF-deficient HSCs [62]. The expression of VEGF and its two receptors Flt-1 and Flk-1 is related to the formation of blood vessels in mouse embryos. VEGF-deficient mice die between E8.5 and 9.5 [63, 64]. Loss of a single VEGF allele is also lethal in the mouse embryo between E11.5 and 12.5. Angiogenesis and blood-island formation were impaired in VEGF heterozygous mouse embryos [63, 64]. Collectively, bone and blood vessel formation is a major component for HSC lodgment into the bone marrow from the fetal liver in mouse (Figure 3).

## 4. Regulating Migration of Fetal Liver Hematopoietic Stem Cells into the Fetal Bone Marrow

### 4.1. Extrinsic Factors

It is well known that SDF-1*α*/CXCR4 signaling is important during the mobilization of both fetal and adult HSCs in mice [63, 65, 66]. Expression of SDF-1*α* is dynamically changed during mouse embryonic development. For example, there are higher expression levels of SDF-1*α* in the E17.5 fetal bone marrow than that in the E14.5 fetal liver and AGM. In response to SDF-1*α*, HSCs from the E14.5 fetal liver and E17.5 bone marrow have much stronger mobilization abilities than HSCs from the E11.5 fetal liver. In comparison to wild-type embryos, deletion of SDF-1*α* leads to severe reduction of HSC numbers in the E18.5 mouse fetal bone marrow along with an increase of HSCs in peripheral blood and spleen [67]. These data indicate that SDF-1*α* expression is required for HSC migration from the mouse fetal liver into the bone marrow.

SDF-1*α* has synergistic effects with other molecules on HSC mobilization abilities. Previous studies have demonstrated that stem cell factor (SCF) or steel factor (SLF) regulates the migration and retention of hematopoietic progenitors through their chemotactic activity in the mouse fetal liver, which is enhanced by SDF-1*α* treatment. These data suggest that SCF and SLF are important factors in HSC homing and seeding to the fetal hematopoietic tissues [64]. Even though expression of CXCR4, an SDF-1*α* receptor, does not dynamically change during different developmental stages in mouse, CXCR4 cooperates with Robo4 to accelerate transplanted HSCs homing and lodging into the bone marrow [68]. This phenomenon is supported by the study using Robo4 knockout mice. Robo4 deletion was compensated by CXCR4 upregulation to maintain the interaction between HSC and bone marrow niches [69, 70]. Robo4 is the predominant Robo receptor on hematopoietic cells. Robo4, but not Robo1, 2, and 3, is highly expressed in HSCs. Moreover, expression of Robo4 on HSCs is dynamically changed. There are much higher levels of Robo4 expression in E17.5 fetal bone marrow HSCs than those in E14.5 fetal liver HSCs. Robo4 expression is further increased when HSCs mature into the adult stage [71]. Therefore, Robo4 might be involved in HSC homing and lodging into the fetal bone marrow from the fetal liver at mid to later gestation, which needs to be further confirmed using genetic mouse models. Other important mediators involving the interaction between HSC and niches are extracellular matrix and adhesion molecules. As discussed above, one of the critical elements controlling fetal HSC mobilization is SDF-1*α*/CXCR4 signaling [72]. This is because SDF-1*α*/CXCR4 signaling regulates extracellular matrix assembly and degradation, mediated by proteins such as MMP2 and MMP9, which is also evidenced in smooth muscle cells and human CD34^+^ progenitors [73]. AMD3100 inhibits SDF-1*α* activity, which leads to bone marrow HSC migration into the spleen and peripheral blood. The process is mediated by MMP9 matrix degradation [74]. It has been reported that MMP2 cleaved SDF-1*α* at locations 4 and 5, resulting in loss of binding ability to CXCR4 on HSCs [75, 76]. G-CSF and SCF increase MMP2 expression along with enhancing bone marrow HSC mobilization into the circulation. Notably, expression of MMP2 in E17.5 fetal bone marrow HSCs is higher than that in E14.5 fetal liver HSCs. This suggests that there might be similar mechanisms for the mobilization of fetal liver HSCs into the bone marrow. High levels of MMP2 in the fetal liver is prepared for HSC traveling toward the circulation and bone marrow. Through quantitative PCR array, Ciriza et al. demonstrated that collagen type IV alpha 1 chain (Col4a1) expression was significantly higher in E17.5 fetal bone marrow LSK cells than in E14.5 fetal liver LSK cells [71]. Col4a1 is a major component in the basement membrane. Tissue inhibitor of metalloproteinase 2 (Timp2) regulates the balance between synthesis and degradation of MMP2 and collagen IV [71]. However, it is unknown how the levels of collagen IV and MMP2 regulate the mobilization of fetal liver HSCs into the fetal bone marrow.

**Figure 4:**
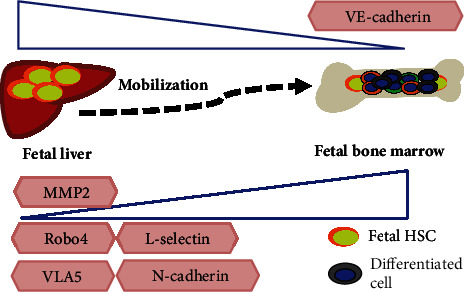
Adhesion molecules regulate fetal liver HSC mobilization into the fetal bone marrow. Increasing levels of MMP2, Robo4, L-selectin, VLA5, and N-cadherin in fetal bone marrow HSCs are involved in HSC mobilization from the fetal liver. Decreasing VE-cadherin expression in fetal HSCs favors HSC mobilization from the fetal liver.

There are a number of adhesion molecules involved with HSC mobilization and homing, such as cadherins, integrins, and selectins (Figure 4). There are two major cadherins involving functional HSCs. Expression of N-cadherin on HSCs is the highest in the E17.5 fetal bone marrow when compared to the E14.5 fetal liver and adult bone marrow, implying that N-cadherin is an initiator of lodging fetal liver HSCs into the bone marrow [67]. VE-cadherin is expressed in both endothelial cells and fetal HSCs, but not adult HSCs [77]. It remains to be known how important VE-cadherin is during the hematopoietic ontogeny.

Compared to cadherins, investigators have extensively explored the functional role of integrin on HSCs. Mutation of *β*1-integrin leads to early embryonic death along with the failure migration of HSCs into the fetal liver and spleen. Similarly, adult *β*1-integrin^−/−^ HSCs remain in the circulation and fail to engraft into the bone marrow niche in recipient mice [78]. These data suggest that *β*1-integrin is a crucial factor in the mobilization and lodgment of HSCs during hematopoietic development. Expression of *α*4*β*1 integrin (VLA-4), *α*5*β*1 integrin (VLA-5), and integrin leukocyte function antigen-1 (LFA-1) has different kinetics during hematopoietic development. Among the fetal liver, fetal bone marrow, and adult bone marrow, VLA-5 expression is the highest in E17.5 bone marrow HSCs while there are comparable levels of VLA-4 and LFA-1 during various hematopoietic periods [79]. These data insinuate that VLA-5 might be implicated during the mobilization of HSCs from the fetal liver into the bone marrow.

Adhesion protein selectins mediate the interactions between endothelial cells and HSCs. The selectins related to HSCs include L-selectin (SELL), E-selectin (SELE), and P-selectin (SELP). SELL expression is higher in E17.5 bone marrow HSCs than in E14.5 fetal liver HSCs. This indicates that SELL might have a certain role in the journey of HSCs from the fetal liver into the bone marrow. Both SELE and SELP are expressed on vascular endothelial cells. Transplanted HSCs failed to engraft into SELE and SELP knockout recipients. The function of selectins on HSCs cooperates with integrins. For example, the failure of HSC engraftment on SELE/SELP mutant mice could be exaggerated under blocking VLA-4 ligand VCAM-1 [80, 81]. Therefore, adhesion molecules regulate migration, homing, and lodgment of HSCs during hematopoietic development.

### 4.2. Intrinsic Factors

**Figure 5:**
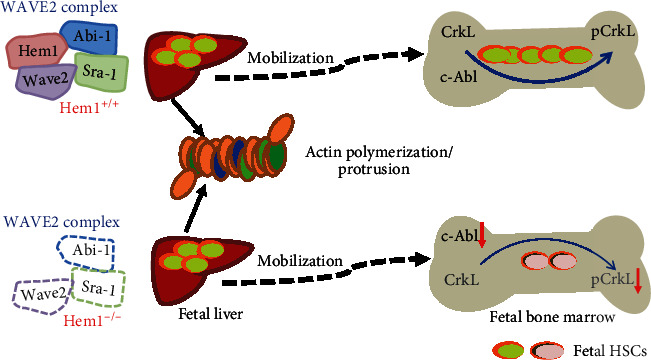
WAVE2 complex regulates fetal liver HSC mobilization into the bone marrow by modulating c-Abl signaling. Loss of Hem1 leads to degradation of WAVE2 complex without affecting actin polymerization in fetal liver HSCs. Lodgment of fetal liver HSCs into the bone marrow is impaired under Hem1 mutation, which is related to inhibiting c-Abl signaling pathway activation with decreasing phosphorylated CrkL (pCrkL).

As we discussed above, SDF-1*α*/CXCR4, extracellular matrix protein, and adhesion molecules regulate the mobilization of fetal liver HSCs into bone marrow niches. However, how intracellular protein in HSCs tailors the transition from the fetal liver into the bone marrow has been limitedly documented. Recently, we investigated the function of Hem1 on fetal hematopoietic stem cell migration [82] (Figure 5). Hem1 protein is one of the members in the WAVE2 complex, which is composed of the Abi-1, Sra-1, Hem-1, and Wave2 proteins. WAVE2 complex has functions in cell migration and actin polymerization [83–85]. There are comparable numbers of HSCs in both E14.5 Hem1^+/+^ and Hem1^−/−^ fetal livers. However, much less fetal bone marrow HSCs in Hem1^−/−^ embryos were observed when compared to those in Hem1^+/+^ ones [82]. Consistently, Hem1^−/−^ fetal liver HSCs failed to rescue lethally *γ*-ray-irradiated mice. These data suggest that Hem1 is required for the transition of HSCs from the fetal liver into the bone marrow. Further studies showed that Hem1 deletion resulted in degradation of the WAVE2 complex with downregulation of Abi-1 and Sra-1 protein, leading to HSC apoptosis [82]. Deletion of Hem1 does not affect actin polymerization and polarization in fetal liver HSCs but inactivates c-Abl signaling and contributes to an HSC lodging deficiency into the bone marrow niche [82]. These findings indicate that application of c-Abl inhibitors to treat diseases, such as leukemia, should be carefully considered during pregnancy. The function of Hem1 is different from Wave2, Rac1, and Rac2. Embryos with depletion of Wave2 or Rac1 or Rac2 died around embryonic day 12.5 due to HSC defects in cellular mobilization and actin polymerization [86, 87]. Therefore, Hem1^−/−^ mice are a perfect mouse model to investigate the importance of the WAVE2 complex in the transition of HSCs from the fetal liver into the bone marrow.

It is well documented that deletion of SDF-1*α*/CXCR4 or SCF/c-Kit did not affect HSC numbers in the E14.5 fetal liver while numbers of HSCs in the E18.5 fetal bone marrow were drastically reduced. In adult mice, inhibiting SDF-1*α* by AMD3100 causes HSC mobilization from the bone marrow into peripheral blood [74]. These data indicate that SDF-1*α* and SCF are required for HSC retention in the bone marrow niche. The next question will be what intracellular downstream factors mediate the responses to SDF-1 and SCF. Recently, Costello et al. provided evidence showing that serum response factor (SRF) might be a candidate responding to SDF-1 and SCF [88, 89]. SRF regulates cytoskeletal and proliferative gene expression in response to extracellular and adhesive signaling [90]. SRF can interact with either the MAP-kinase-regulated ternary complex factors (TCFs) or the G-actin-regulated myocardia-related transcription factors (MRTF-A and MRTF-B) [91, 92]. Loss of TCFs does not affect the reconstitution ability in fetal liver HSCs while MRTF-SRF signaling plays an important role in platelet function and megakaryocyte differentiation. Knockout of SRF or MRTFs leads to perinatal death without affecting HSC function in the E14.5 fetal liver [89]. This phenotype is very similar to the depletion of Hem1, SDF-1*α*, or SCF. These data hint that MRTF-SRF signaling plays a critical role in the HSC transition from the fetal liver into the bone marrow. In response to SDF-1*α* or SCF, mutation of SRF or MRTFs in the E14.5 fetal liver exhibited multiple defective behaviors in adhesion and mobilization, which lead to the impairment of homing and engraftment capacities under transplantation settings using SRF^−/−^ or MRTFs^−/−^ fetal liver HSCs. However, the underlying mechanism of MRTF-SRF signaling in fetal liver HSCs is different from that of WAVE2 complex protein Hem1. Under the transition of HSCs from the fetal liver to the bone marrow, loss of Hem1 shuts down c-Abl survival signaling in the bone marrow niche without affecting fetal HSC polarization. However, deletion of SRF or MRTFs negatively affects mobilization and lodgment of HSCs into the bone marrow niche. In both scenarios, they will result in hypocellularity in the fetal bone marrow and embryonic death. Therefore, both extrinsic and intrinsic factors modulate the transition of HSCs from the fetal liver into the bone marrow.

## 5. Conclusion

Hematopoietic stem cells are rapidly expanded in E12.5 to E15.5 fetal livers in mice. Both cell autonomous and noncell autonomous factors regulate HSC expansion in the fetal liver. What potential mechanisms are involved in the expansion of fetal liver HSCs is an interesting and important question. We might find simple approaches to *in vitro* expand HSCs for clinical transplantation if we deeply understand the underlying expansion mechanisms. Thus far, we already knew that the proliferation of Nestin^+^NG2^+^ pericytes around portal vessels was a critical determinant for fetal HSC expansion. Mutation of NG2 could not completely block fetal HSC expansion, indicating that other factors from fetal livers might be involved in the expansion. However, it is unknown whether endothelial and hematopoietic cells regulate fetal liver HSC expansion. There is also a need to define which signaling pathways from endothelial and hematopoietic cells accelerate fetal liver HSC expansion. They, most likely, play some roles in the expansion. This is because endothelial cells, B cells, and megakaryocytes were reported as niches for adult bone marrow hematopoietic stem cells. Another possibility is that fetal hepatocytes, a dominant cell type in the fetal liver, regulate fetal HSC expansion through direct and indirect mechanisms. It has been visualized that EC “pockets” in CHT in zebrafish and Nestin^+^NG2^+^ cells in the fetal liver in mouse were associated with HSCs. However, the spatial localization of fetal liver niche cells (such as hepatocytes and endothelial and stromal cells) with fetal HSCs in mammals needs to be defined. This will be helpful to understand how fetal niche cells regulate fetal HSC function.

It seems that both vascularization and endochondral ossification are required for fetal HSC lodging into the bone marrow. So far, rare intrinsic factors in HSCs are identified to be responsible for fetal HSC mobilization into the bone marrow. What factors from endothelial and bone elements regulate fetal liver HSC mobilization into the fetal bone marrow? Understanding these unanswered questions will help us to modify the bone marrow environment in the transplantation settings. Understanding components regulating fetal HSC mobilization into the bone marrow will guide us to prepare clinical therapeutic protocols for leukemia treatment during pregnancy, avoiding negatively affecting the fetal hematopoietic system.

## Figures and Tables

**Figure 1:**
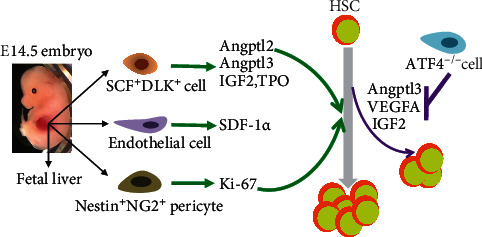
Microenvironment in the fetal liver is crucial for HSC proliferation. SCF ^+^ DLK ^+^ cells secret Angptl2, Angptl3, IGF2, and TPO to accelerate HSC proliferation in the fetal liver. Nestin^+^NG2^+^ pericytes associated with portal vessels is a fetal liver niche, supporting HSC expansion. Fetal liver endothelial cell produces SDF-1*α* favoring HSC mobilization. Loss of ATF4 in the fetal liver niche reduces HSC expansion through decreasing Angptl3, IGF2, and VEGFA.

## Abbreviations

AGM: Aorta-gonad mesonephros
Angptl2: Angiopoietin-like 2
Angptl3: Angiopoietin-like 3
ATF4: Activating transcription factor 4
Ccl25b: Chemokine ligand 25b
CD105^+^Thy1^−^: CD452^−^Tie2^−^aV^+^CD105^+^1Thy1.1^−^
CHT: Caudal hematopoietic tissue
Cited 2: cAMP-responsive element binding protein [CBP]/p300-interacting transactivators with glutamic acid [E] and aspartic acid [D]–rich tail 2
Col4a1: Collagen type IV alpha 1 chain
CVP: Caudal vein plexus
EC: Endothelial cells
EHT: Endothelial cell to hematopoietic cell transition
ER: Endoplasmic reticulum
ESAM: Endothelial cell-selective adhesion molecule
HSCs: Hematopoietic stem cells
IAC: Intra-arterial clusters
IGF-2: Insulin-like growth factor 2
Klf6a: Kruppel-like factor 6a
LFA-1: Leukocyte function antigen-1
IL-1*β*: Interleukin-1-beta
MAZ: Myc-associated zinc finger
MMP2: Matrix metallopeptidase 2
MMP9: Matrix metallopeptidase 9
MRTFs: Myocardia-related transcription factors
N-cad: N-cadherin
Nothc1-TAD: Notch1 transcriptional activation domain
Notch1*Δ*/TAD: Loss one copy of Notch1-TAD
scRNA-Seq: Single-cell RNA sequencing
scATAC-Seq: Single-cell assay for transposase-accessible chromatin sequencing
SRF: Serum response factor
SLF: Steel factor
SCF: Stem cell factor
SELL: L-Selectin
SELE: E-Selectin
SELP: P-Selectin
TCFs: Ternary complex factors
TPO: Thrombopoietin
Timp2: Tissue inhibitor of metalloproteinase 2
VCAM-1: Vascular cell adhesion molecule 1
VLA-4: *α*4*β*1 integrin
VLA-5: *α*5*β*1 integrin.
